# The Feasibility of Omitting Postoperative Radiotherapy in Japanese Patients With Ductal Carcinoma In Situ of Breast Treated With Breast-Conserving Surgery

**DOI:** 10.7759/cureus.48187

**Published:** 2023-11-02

**Authors:** Akihiro Nakashima, Hideya Yamazaki, Gen Suzuki, Kei Yamada, Aibe Norihiro, Takuya Kimoto, Koji Masui, Katsuhiko Nakatsuka, Tetsuya Taguchi, Yasuto Naoi

**Affiliations:** 1 Radiology, Kyoto Prefectural University of Medicine, Kyoto, JPN; 2 Radiology, Graduate School of Medical Science, Kyoto Prefectural University of Medicine, Kyoto, JPN; 3 Endocrine and Breast Surgery, Kyoto Prefectural University of Medicine, Kyoto, JPN

**Keywords:** omitting radiotherapy, ductal carcinoma in situ, breast conserving surgery, postoperative radiotherapy, breast cancer

## Abstract

Background

To analyze the feasibility of omitting postoperative radiotherapy (PORT) after breast-conserving surgery (BCS) in Japanese patients with ductal carcinoma in situ (DCIS).

Materials and methods

We retrospectively analyzed 88 patients with small pure DCIS (median diameter 1.1 cm, ≤ 4 cm) who underwent BCS with (n = 39) or without (n = 49) PORT. The primary and secondary endpoints were ipsilateral breast tumor recurrence (IBTR) and overall survival (OS), respectively, between the groups that received PORT and those that did not.

Results

The PORT group included a high number of margin-positive cases. The incidence of IBTR was 2.4% (95% confidence interval (CI), 0.3-15.7%) and 2.8% (95% CI, 0.4-18.2%) at five years and 5.5% (95% CI, 1.4-20.6%) and 2.8% (95% CI, 0.4-18.2%) at 10 years in patients without and with PORT, respectively (p = 0.686). In the margin-negative group, only one patient showed IBTR without RT (2.3%), whereas no patient with PORT experienced IBTR (0%). To date, there have been no regional or distant metastases; therefore, no patient has experienced breast cancer-related deaths. The OS rates were 97.7% (95% CI, 84.9-99.6%) and 100% at 10 years in patients without and with PORT, respectively (p = 0.372).

Conclusion

This study suggests that the omission of PORT after BCS could be a feasible option for selected Japanese patients but requires further investigation to identify the low-risk factor in patients who can omit PORT.

## Introduction

Breast cancer in women has emerged as the most commonly diagnosed cancer worldwide, with approximately 2.3 million new cases estimated in 2020 [1]. Of these, ductal carcinoma in situ (DCIS) represents approximately 20% of the cases in the USA [2]. Postoperative radiotherapy (PORT) is the established standard treatment after breast-conserving surgery (BCS) for DCIS, and mastectomy is reserved for diseases extending beyond one quadrant. Several randomized clinical trials and meta-analyses have shown that PORT reduces local recurrence by approximately 50% [3-7], without improving the metastasis-free or overall survival (OS) rates.

To avoid PORT side effects, such as breast pain, dermatitis, and the risk of heart and lung complications, including radiation-induced secondary cancer [8], the omission of PORT was explored to identify a lower-risk group, that is, clinicopathological features (CPF), such as age at diagnosis, lesion size, nuclear grade, comedonecrosis, multifocality, and margin width, partly due to the limited benefits of PORT [9]. Although several retrospective and prospective trials have been performed in Western countries to omit PORT, the optimal conditions for omitting PORT have not yet been established [9-12]. In addition, there is limited evidence available in Asian patients, although there are differences in the efficacy and toxicity of systemic therapy [13]. Therefore, we retrospectively reviewed our database.

 The objective of this study was to analyze the impact of PORT in Japanese patients with small DCIS of the breasts treated with BCS.

## Materials and methods

We retrospectively examined the data of patients who underwent surgery for clinically diagnosed breast DCIS at the Department of Endocrine and Breast Surgery, Kyoto Prefectural University of Medicine. Data from 179 patients were obtained between January 2001 and May 2016.

We included histologically confirmed DCIS of the breast with available and accessible data on age, histology, hormonal status (ER, PgR, and HER2), information for systemic therapy, and radiotherapy. Of these, we excluded patients with microinvasion, lobular carcinoma in situ, positive lymph nodes with a pathological examination of the surgical specimen, short follow-up of less than six months without any failure, incomplete radiotherapy before the planned dose, and total mastectomy. We retrospectively analyzed 88 patients who underwent BCS with and without PORT (n = 49 vs. n = 39).

PORT was administered at 50 Gy in a daily 2 Gy fraction in two opposing tangential fields to the residual breast tissue using 6-MeV photons. Some cases in which the margin status was positive on pathology received an additional 10 Gy in five fractions using electron beams. Margin positive is generally defined as < 2 mm.

The primary endpoint was ipsilateral breast tumor recurrence (IBTR), which was defined as any recurrence, including invasive carcinoma and DCIS, in the ipsilateral irradiated breast. The second endpoint was the OS rate. We compared the IBTR and OS between the groups that received PORT or did not receive PORT.

All patients were enrolled in the study after obtaining informed consent before undergoing BCS, according to the guidelines of the Institutional Ethics Committee of the Kyoto Prefectural University of Medicine (IRB approval number: ERB-C-1363-1).

Statistical analyses

The EZR Stat packages (https://www.jichi.ac.jp/saitama-sct/SaitamaHP.files/statmedEN.html) were used for statistical analyses [14]. Percentages were analyzed using Fisher’s exact test, and the student’s t-test was used for normally distributed data. The Mann-Whitney U-test was used to compare skewed data. The Kaplan-Meier method was used to analyze the LC and OS, which were compared using the log-rank test. The time to event was determined from the day of surgery. Cutoff values were set as the median or mean values unless otherwise specified. Statistical significance was set at p < 0.05.

## Results

Patients’ characteristics and influence of PORT

Median follow-up time was 6.7 years (range: 6 months-13.8 years). Table 1 lists patient and treatment characteristics. The PORT group consisted of patients with marginally positive lesions.

**Table 1:** Patient characteristics *Comparison was made excluding the unknown category ER: estrogen receptor, PgR; progesterone receptor, HER2: human epidermal growth factor receptor 2, PORT; postoperative radiotherapy

Factor	Group	Total n = 88 No. (%) or Median (range)	PORT (-) n = 49 No. (%) or Median (range)	PORT (+) n = 39 No. (%) or Median (range)	p-value
Age		52.00 (28.00, 88.00)	52.00 (28.00, 88.00)	51.00 (32.00, 82.00)	0.223
Tumor diameter	(cm)	1.10 (0.00, 4.00)	1.40 (0.00, 3.50)	1.00 (0.00, 4.00)	0.891
ER	Negative	32 (36.4)	19 (38.8)	13 (33.3)	0.659
	Positive	56 (63.6)	30 (61.2)	26 (66.7)	
PgR	Negative	28 (31.8)	10 (28.6)	18 (48.6)	*0.095
	Positive	44 (50.0)	25 (71.4)	19 (51.4)	
	Unknown	16 (18.2)	14 (28.6)	2 (5.1)	
HER2	Negative	61 (69.3)	32 (97.0)	29 (85.3)	*0.197
	Positive	6 (6.8)	1 (3.0)	5 (14.7)	
	Unknown	21 (23.9)	16 (32.7)	5 (12.8)	
Nuclear grading	0	7 (8.0)	4 (8.2)	3 (7.7)	*0.538
	1	5 (5.7)	4 (8.2)	1 (2.6)	
	2	38 (43.2)	17 (34.7)	21 (53.8)	
	3	15 (17.0)	7 (14.3)	8 (20.5)	
	Unknown	23 (26.1)	17 (34.7)	6 (15.4)	
Margin	Negative	61 (70.1)	44 (89.8)	17 (44.7)	<0.001
	Positive	26 (29.9)	5 (10.2)	22 (56.4)	
Preoperative Systemic Therapy	No	83 (94.3)	46 (93.9)	37 (94.9)	1
	Yes	5 (5.7)	3 (6.1)	2 (5.1)	
Adjuvant Systemic Therapy	No	59 (67.0)	33 (67.3)	26 (66.7)	1
	Yes	29 (33.0)	16 (32.7)	13 (33.3)	

**Figure 1:**
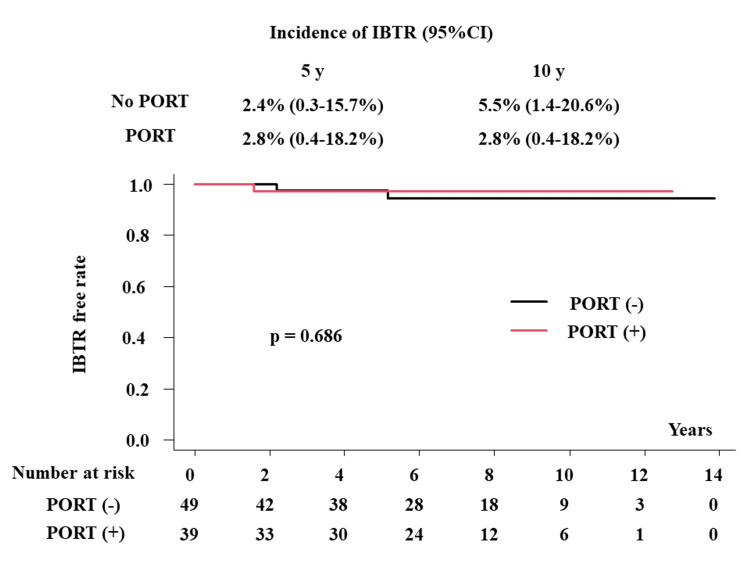
The ipsilateral breast tumor recurrence-free rate with or without postoperative radiotherapy PORT: postoperative radiotherapy, IBTR: ipsilateral breast tumor recurrence

The local control rates were 97.6% (95% confidence interval (CI), 84.3-99.7%) and 97.2% (95% CI, 81.8-99.6%) at five years and 94.5% (95% CI, 79.4-98.6%) and 97.2% (95% CI, 81.8-99.6%) at 10 years in patients with and without PORT, respectively (p = 0.686, Figure 1). Therefore, the incidence of IBTR was 2.4% (95% CI, 0.3-15.7%) and 2.8% (95% CI, 0.4-18.2%) at five years and 5.5% (95% CI, 1.4-20.6%) and 2.8% (95% CI, 0.4-18.2%) at 10 years in patients without and with PORT (p = 0.686, Figure 1).

In the margin-negative group (n = 61), one patient showed IBTR without RT (1/44, 2.3%), whereas no patient with PORT experienced IBTR (0/17, p =1.0). In the margin-positive group (n = 27), one patient showed IBTR (1/5 = 20%) while one patient experienced IBTR in patients with PORT (1/22 = 4.5%, p = 0.342). No factors influencing IBTR were identified (Table 2).

**Table 2:** Analysis of influential factors for IBTR *Comparison was made excluding unknown category ER: estrogen receptor, PgR; progesterone receptor, HER2: human epidermal growth factor receptor 2; PORT; post-operative radiotherapy, IBTR: ipsilateral breast tumor recurrence

Factor	Group	No IBTR (n = 85) No. (%) or Median (range)	IBTR (n = 3) No. (%) or Median (range)	p-value
Age		52.00 (28.00, 88.00)	50.00 (40.00, 65.00)	0.687
	23-49	34 (40.0)	1 (33.3)	0.699
	50-59	29 (34.1)	1 (33.3)	
	60-69	13 (15.3)	1 (33.3)	
	70-79	7 (8.2)	0 (0.0)	
	80-89	2 (2.4)	0 (0.0)	
Tumr diameter	(cm)	1.20 (0.00, 4.00)	0.40 (0.00, 0.80)	0.163
ER	Negative	31 (36.5)	1 (33.3)	1
	Positive	54 (63.5)	2 (66.7)	
PgR	Negative	28 (32.9)	0 (0.0)	*0.427
	Positive	42 (49.4)	2 (100.0)	
	Unknown	15 (17.6)	1 (33.3)	
HER2	Negative	60 (70.6)	1 (33.3)	*0.105
	Positive	5 (5.9)	1 (50.0)	
	Unknown	20 (23.5)	1 (33.3)	
Nuclear grading	0	7 (8.2)	0 (0.0)	*0.733
	1	5 (5.9)	0 (0.0)	
	2	36 (42.4)	2 (66.7)	
	3	14 (16.5)	1 (33.3)	
	Unknown	23 (27.1)	0 (0.0)	
Margin	Negative	60 (70.6)	1 (33.3)	0.222
	Positive	25 (29.4)	2 (66.7)	
PORT	No	47 (55.3)	2 (66.7)	1
	Yes	38 (44.7)	1 (33.3)	
Preoperative Systemic Therapy	No	81 (95.3)	2 (66.7)	0.163
	Yes	4 (4.7)	1 (33.3)	
Adjuvant Systemic Therapy	No	57 (67.1)	2 (66.7)	1
	Yes	28 (32.9)	1 (33.3)	

**Figure 2:**
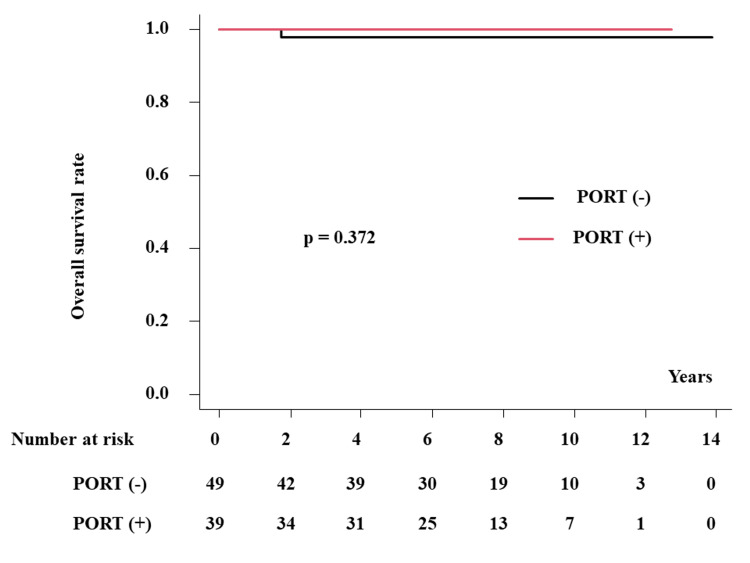
The overall survival rate with or without postoperative radiotherapy PORT: postoperative radiotherapy

No recurrence events were identified in the regional lymph nodes or distant sites; therefore, no breast cancer-related deaths occurred. The OS rates were 97.7% (95% CI, 84.9-99.6%) and 100% at five years and 97.7% (95% CI, 84.9-99.6%) and 100% at 10 years in patients with and without PORT, respectively (p = 0.372, Figure 2). 

Three patients developed IBTR after undergoing PORT. A 40-year-old patient with a positive margin (close margin: 1 mm) who received 60 Gy in 30 fractions of PORT developed IBTR at the original site after 18.8 months. The maximum diameter of the clinical tumor extension at the initial treatment was 0.8 cm (ER-positive, PgR-positive, HER2-negative, nuclear grade 2). The patient underwent salvage BCS, and the pathological diagnosis of the IBTR was invasive carcinoma. The patient is alive without any evidence of a tumor 122 months later. The other patient with IBTR was 65 years old. She underwent neoadjuvant systemic therapy (Letrozole → Taxol + Herceptin) depending on the histology of the biopsy specimen (hormonal therapy and ER-positive, PgR positive, HER2 positive, nuclear grade 3) and achieved a good tumor response (Figure 2). The histological findings revealed a positive margin (2 mm); however, it was in the direction of the skin, thereby omitting PORT, and the patient underwent adjuvant treatment (hormonal therapy and anti-HER agents). She developed IBTR at the original site after 25.6 months and underwent a salvage mastectomy. The patient was alive without any evidence of a tumor 91.3 months later. The third patient was 50 years old, and she had a small DCIS (negative margin, ER-negative, nuclear grade 2) without PORT. Local recurrence appeared 61.9 months later, and the patient was successfully treated and alive with no recurrence 126 months later.

## Discussion

We examined the effects of PORT after BCS in Japanese patients with breast DCIS. We showed good outcomes of BCS with or without PORT (5.5% and 2.8% of IBTR at 10 years in patients with and without PORT, respectively) but did not affect the OS rate. In the margin-negative group (n = 61), one patient showed IBTR without RT (1/44, 2.3%), whereas no patient with PORT experienced IBTR (0/17, p =1.0). In the margin-positive group (n = 27), one patient showed IBTR (1/5 = 20%) while one patient experienced IBTR in patients with PORT (1/22 = 4.5%, p = 0.342).

There are two possible reasons for this good results. At first, appropriate patient selection may be a major reason for good results. Our patient had a relatively small unicentric disease (median diameter 1.1 cm) with majorly ER-positive and a negative margin. Next, there may be an ethnic reason. There are inherent and potentially significant differences between Caucasian and Asian patients with breast cancer, reflecting differences in host biology, socioeconomic realities, lifestyle, health-seeking behaviors, and treatment effects. Japanese patients generally showed superior results for systemic agents [15,16].

DCIS is defined as the malignant proliferation of cells within the breast ducts. Mastectomy is mainly performed in patients with diffuse infiltrative disease, large tumors, or involved surgical margins after repeated resection [17]. In our study, 30 of 179 (16.7%) patients underwent mastectomy because of the nature of the diseases mentioned above. BCS followed by PORT is an acceptable treatment for small and unifocal areas of DCIS; however, there is insufficient evidence to omit PORT routinely [9-11].

Four randomized trials have demonstrated that irradiation reduces the risk of IBTR after DCIS excision from approximately 15% to 20% at five years and from 25% to 30% at 10 years to approximately 5% to 9% and 15%, respectively, in relatively unselected patients [3-6]. The Early Breast Cancer Trialists' Collaborative Group made a meta-analysis using those RCTS and reported that PORT reduced the absolute 10-year risk of IBTR by 15.2% (12.9% vs. 28.1%, P <.00001) [7]. Our data showed that the incidence of IBTR was 5.5% (1.4-20.6%) and 2.8% (0.4-18.2%) at 10 years in patients with and without PORT, respectively, which seems to be a better outcome and partially concurs with these data. 

In addition, these trials did not show an improvement in distant metastasis-free survival, breast cancer-specific survival, or OS [3-7], in contrast to the results of trials for invasive cancer, where PORT could improve these outcomes [18]. Hence, the toxicity of PORT should be considered more carefully when making treatment decisions in patients with DCIS than in those with invasive carcinoma. Several investigators have explored the omission of PORT and reported IBTR rates of 5%-15% at 5-10 years in selected patients treated with local excision alone with wide margins [9-12].

McCormick et al. reported the long-term results of RTOG 9804 [12]; that at 13.9, a median follow-up, the 15-year cumulative incidence of IBR was 7.1% with PORT versus 15.1% without PORT. In this study, low-risk DCIS was defined as unicentric disease, tumor size < 2.5 cm, low or intermediate nuclear grade, or negative surgical margins of 3 mm or greater from a lumpectomy specimen. In contrast, several studies have observed a high rate of local recurrence in a small group of low-to-intermediate-risk patients with DCIS who did not receive radiotherapy [9-12]. Similarly, Motwani et al. cautioned against withholding adjuvant radiotherapy in low-risk patients [19].

Shikama et al. conducted a prospective study for high-risk DCIS using PORT resulted in a five-year IBTR rate of 6%, and this schedule is promising for patients with a single DCIS lesion with an involved surgical margin or close margin widths of ≤ 1 mm [20]. Our data also concurred with the outcome that the PORT group showed 2.8% IBTR, including 55% of margin-positive patients, which is generally defined as < 2 mm. Dunne et al. conducted a systematic review and reported that a margin threshold of 2 mm seemed to be as good as a larger margin when BCS for DCIS was combined with PORT [21].

Molecular profiling is a new approach to defining risk stratification for DCIS, and two tissue-based assays for assessing DCIS IBR risk are commercially available in the United States [22]. After lumpectomy for DCIS, risk stratification using clinical and pathological characteristics, and more recently, molecular profiling can help guide physicians and patients in the use of PORT and hormonal therapy.

This study has several limitations. First, our data were derived from a retrospective study of a small population with limited follow-up periods and a possible selection bias. Next, detailed information on systemic therapy, histological findings, nuclear grading, and hormonal receptors was lacking for several patients, although this did not influence the conclusion. However, large-scale randomized trials are required to draw definitive conclusions.

## Conclusions

This study suggests that the omission of PORT after BCS could be a treatment option for Japanese patients with small DCIS with a negative margin. This study suggests that the omission of PORT after BCS could be a feasible option for selected Japanese patients and requires further prospective study to confirm the low-risk group who can omit PORT. These data will help inform the decision-making processes of patients and their physicians.
